# A case of fibromyalgia involving pain throughout the body treated with site-specific targeted pain control

**DOI:** 10.1186/s40064-016-2572-z

**Published:** 2016-07-08

**Authors:** Fukami Nakajima, Satoko Aratani, Hidetoshi Fujita, Kou Nakatani, Koshi Makita, Toshishiro Nakajima

**Affiliations:** Daiichi Rehabilitation Hospital, 2-14 Kutanda Kochi-shi, Kochi, 781-0112 Japan; Department of Locomotor Science, Institute of Medical Science, Tokyo Medical University, 6-1-1, Shinjuku, Shinjuku-ku, Tokyo, 160-8402 Japan; Physician, Student, and Researcher Support Center, Tokyo Medical University, Tokyo, Japan; Future Medical Science Institute of Medical Science, Tokyo Medical University, Tokyo, Japan; Bayside Misato Medical Center, Kochi, Japan; Department of Anesthesiology, Graduate School of Medical and Dental Sciences, Tokyo Medical and Dental University, Tokyo, Japan

**Keywords:** Fibromyalgia, Musculoskeletal pain, Nerve block

## Abstract

**Introduction:**

Fibromyalgia is characterized by chronic pain and tenderness throughout the body. Patients with fibromyalgia are treated with pharmacotherapy and many other therapies. However, because the cause of fibromyalgia is unclear, there is currently no clinically effective treatment method.

**Case presentation:**

We report the case of a patient who developed fibromyalgia after left femoral neck fracture. After several caudal epidural blocks for lumbar pain, the pain throughout the body and abnormal discomfort in the laryngopharyngeal region reduced. Site-specific targeted pain control was effective in treating his pain and discomfort.

**Conclusion:**

The present case suggests that treatment targeting symptoms in one part of the body might produce a systemic therapeutic effect in patients with fibromyalgia.

## Introduction

Fibromyalgia is a disease associated with chronic pain and tenderness throughout the body; however, its etiology is unknown, and it is often accompanied by depression, fatigue, and sleep disorders (Wolfe 1988, 1990; Wolfe et al. 1995). Drug therapy (Imani and Rahimzadeh 2012; Alimian et al. 2012a, b; Hill et al. 2012; Ablin and Buskila 2013), cognitive behavioral therapy (Ablin et al. 2013), exercise therapy, nerve block therapy (Bengtsson and Bengtsson 1988), alternative therapies (Nakajima et al. 2015), and other approaches have been used for the treatment of this disease. However, because the cause of fibromyalgia is unclear, there is currently no clinically effective treatment. The complaints of patients are often indistinct, and in some instances, treatment is agonizing. It is often difficult to treat pain present over the entire body. Herein, we report the effective use of site-specific targeted pain control for treating a range of symptoms throughout the body in a patient who developed fibromyalgia after left femoral neck fracture.

## Case presentation

### Case

The patient was a 42-year-old man (height, 168 cm; weight, 68 kg).

### History of the present illness

At the age of 37 years, the patient collided with a utility pole while riding a bicycle and experienced left femoral neck fracture. He underwent an open reduction and internal fixation procedure under general anesthesia, and both the surgery and postoperative course were uneventful. However, he experienced back pain immediately after the surgery. He later gradually began to experience increasingly severe pain in all body muscles, pain in his joints, and discomfort in the laryngopharyngeal area. Despite multiple hospital visits, the cause of his pain remained unknown, and in April 2013, at the age of 39 years, he was diagnosed with fibromyalgia according to the diagnostic criteria of the American College of Rheumatology (Wolfe et al. 1990) at the outpatient fibromyalgia department of our hospital, which specializes in fibromyalgia treatment. It is sometimes difficult to discriminate fibromyalgia from psychiatry illnesses; however, he was not diagnosed with any psychiatric disorders by the psychiatrist at our hospital. After diagnosis, he received medication (pregabalin 125 mg/day *per os*, rebamipide 300 mg/day *per os*, milnacipran hydrochloride 7.5 mg/day *per os*, and etizolam 0.5 mg/day *per os*) and underwent rehabilitation. For improved symptomatic relief, he visited the outpatient pain department of our hospital in September 2014, followed by combined treatments at the outpatient fibromyalgia and pain departments of our hospital.

### Examination findings at the first visit

At the first visit to the outpatient pain department, the patient had a Widespread Pain Index and Symptom Severity (WPISS) score (Fitzcharles et al. 2012) of 23 points (WPI, 15 points; SS, 8 points), and a score of 750 in assessments with PainVision (Nipro Co., Osaka, Japan) (Kato 2011), a quantitative pain analysis device.

### Course of treatment

At the first visit to the outpatient pain department, the patient complained of severe pain throughout the body. His answer to the question “Where does it hurt most?” was “The worst discomforts are the abnormal sensation in my laryngopharyngeal area and the back pain.” He experienced very severe back pain in the region from Th3 to Th7. Therefore, he was administered trigger point injections (1 mL of 0.5 % lidocaine at each tender point) at the painful areas on his back. At each visit, approximately ten tender locations were treated. He was administered one set of trigger point injections monthly for 4 months, and each time, the pain subsided for several days after treatment but returned unabated after several days. Five months after his first visit to the outpatient pain department, we explained the methodologies, actions, effects, and complications of stellate ganglion block, thoracolumbar epidural block, and caudal epidural block. He selected caudal epidural block, as it has a low risk of complications such as subarachnoid puncture. He underwent caudal epidural block (3 mL of 0.2 % ropivacaine + 6 mL of saline + 1.65 mg of dexamethasone) for his lumbar pain during an outpatient visit. Immediately following the caudal epidural block, not only his lumbar pain, but also his upper back pain was alleviated for approximately 2 weeks. Therefore, he underwent another caudal epidural block at his own request in the sixth month during an outpatient visit. His back pain and the abnormal sensation in the laryngopharyngeal area were alleviated for approximately 3 weeks after the second block. He continued receiving caudal epidural blocks once a month, and after a total of four blocks, his back pain was alleviated for approximately 3 weeks after the block, and the abnormal sensation in the laryngopharyngeal area almost disappeared. After approximately 3 weeks, the pain returned; however, the intensity was much lower than that at the first visit. On assessing his pain using PainVision (Kato 2011), we found that his PainVision score of 750 at the first visit reduced to 139 at the ninth month (1 month after the fourth epidural block). Medication was used as an adjunct therapy at the outpatient fibromyalgia clinic. The patient’s mental condition might have contributed to his symptoms associated with fibromyalgia; however, there was no change in his prescription drug regimen and his mental condition during the period presented in this report.

## Discussion

The degree of pain and abnormal discomfort in our patient remained unchanged for 1 year before his first visit to our pain clinic; moreover, the medication prescription had been the same for the past 6 months. Furthermore, his mental state did not change around the time of nerve block administration. Nevertheless, his pain was significantly alleviated immediately after the nerve block. Based on these findings, we believe that the degree of pain and abnormal discomfort mainly decreased as result of the caudal epidural blocks. We hypothesized that for fibromyalgia patients when pain in one part of the body changes (such as at fibromyalgia onset), it can affect other parts of the body that are not anatomically related to the area of innervation.

The proposed mechanisms for fibromyalgia include failure of the descending pain modulatory system (La Cesa et al. 2014; Macian et al. 2015) and central sensitization (Fleming and Volcheck 2015; Staud et al. 2001); however, its causes are not clear.

According to the Practice Guideline for Fibromyalgia 2013 (edited by the Japan College of Fibromyalgia Investigation), the initial pain in fibromyalgia triggers subsequent pain, and the pain gradually spreads, in no particular order, to areas with no anatomical connection to the area of innervation (Japan College of Fibromyalgia Investigation 2013). As such, fibromyalgia often involves pain in only a part of the body at onset, which then gradually spreads to other parts until pain is felt throughout the body.

In the present case, it was inferred from the interview of the patient that the femoral neck fracture triggered fibromyalgia and that the pain in the femoral region gradually spread throughout the body. It is possible that even after treatment of his femoral neck fracture, cellular firing of posterior horn neurons at the level corresponding to the fracture continued, resulting in central sensitization. However, as the onset mechanism of fibromyalgia is unclear, it is unknown how the femoral pain affected other parts of the body. Additionally, when pain is alleviated, it is possible that pain changes in one part of the body could affect pain in other parts through the same mechanism involved in fibromyalgia onset.

Incorrect information transmission at the central level, such as the thalamus, might be involved in the mechanism of onset of fibromyalgia, and we believe that pain changes in one part of the body might affect pain in other parts of the body both at the time of fibromyalgia onset and after onset. It is not clear whether this hypothesis is accurate. Therefore, additional studies are required to understand the mechanism.

## Conclusions

This case report suggests that a treatment targeting symptoms in one part of the body and the alleviation of the part of the body might produce a therapeutic effect throughout the body in patients with fibromyalgia. Clinicians should not give up on treatment goals, even when there are many target regions presenting symptoms requiring treatment.
